# Prevalence and Perceived Usefulness of Assistive Technology in Mind at Home Dementia Cohort

**DOI:** 10.1093/geroni/igaa057.632

**Published:** 2020-12-16

**Authors:** Michael Morreale, Deirdre Johnston, Morgan Bunting, Inga Antonsdottir, Quincy Samus

**Affiliations:** 1 Johns Hopkins University, Swarthmore, Pennsylvania, United States; 2 Johns Hopkins Hospital, Baltimore, Maryland, United States; 3 Johns Hopkins University, Halethorpe, Maryland, United States; 4 Johns Hopkins University, Baltimore, Maryland, United States

## Abstract

Dementia is generally characterized by both an increasing dependence in activities of daily living over the course of the illness, and a decreasing ability to self-manage everyday tasks. This places persons at risk for a number of undesirable outcomes including increased risk for injury in the home, increased risk for medical and behavioral complications, risk of premature institutionalization, and excessive burden on family caregivers (CG).3,4 Assistive Technology Devices (AT-Devices) could represent an efficient resource for supporting daily tasks while reducing both CG care burden and adverse risk to the person with dementia (PWD).3,4 In the context of a larger dementia care intervention clinical trial (The MIND at Home program) that involved persons living with dementia at home and their family caregivers, we conducted a supplemental baseline survey on 59 participants and their CGs to better understand the current prevalence of AT-Device use and which devices would be perceived as “most helpful”. Our analysis showed that 51% of our study population used at least 1 of our listed AT-Devices. The most common AT-Device used at baseline were door guards (29%), tablets/smartphones (20%), and constant temperature shower nozzles (13%). Our survey demonstrated devices perceived as most useful included: shower nozzles, GPS locating devices, door guards, and Bluetooth tracking stickers. Individuals who endorsed African-American/Other race were significantly more likely to use at least one AT-Device than those who identified as Caucasian (OR: 4.80; 95% CI: 1.50-17.58). This significance was lost during adjustment for other demographic variables (sex, age, cohabitation status, and dementia severity).

